# Management of Old Gustilo-Anderson Type IIIB Open Tibial Fractures With Medial Gastrocnemius or Combined Medial Gastrocnemius-Hemisoleus Flaps

**DOI:** 10.7759/cureus.80687

**Published:** 2025-03-16

**Authors:** Rajib Sarkar, Samriddhi Sarkar, Sayantika Sarkar, Atanu Pramanik, Ujjwal Singh, Sanjay Roy

**Affiliations:** 1 Orthopaedic Surgery, ICARE Institute of Medical Sciences and Research, Haldia, IND; 2 Orthopaedics, Mahatma Gandhi Medical College and Research Institute, Pondicherry, IND; 3 Emergency Medical Services, Sir HN Reliance Foundation Hospital and Research Prarthana Samaj, Mumbai, IND

**Keywords:** gustilo-anderson type iiib, local flap, medial gastrocnemius flap, old gustilo-anderson type iiib, open fracture fixation, open tibial fracture, soleus muscle flap

## Abstract

Introduction

Old Gustilo-Anderson Type IIIB open tibial fractures presenting one to three months post-primary management with deep infection, superinfection, and inadequate soft tissue coverage pose significant treatment challenges. This study evaluates the outcomes of medial gastrocnemius or combined medial gastrocnemius-hemisoleus flaps in managing these old injuries, along with adjuvant orthopaedic (orthoplastic) procedures.

Methods

A retrospective review was conducted on 15 cases involving fractures of the middle third of the tibia. These patients had initially undergone debridement and external fixation as primary management and presented one to three months post-injury. Management involved infection control through daily irrigation and debridement. In the definitive stage, revision of external fixation was performed along with decortication of the exposed tibia, bone grafting, and local muscle flap coverage.

Results

All fractures achieved union with complete infection eradication and durable soft tissue coverage, enabling full functional recovery with normal gait and strength. All patients returned to their pre-injury functional status and occupations.

Discussion

Old Type IIIB fractures are underreported. This study highlights the role of staged infection control, decortication of the exposed tibia, bone grafting, revision of external fixation, and local muscle flap coverage in promoting bone healing, soft tissue restoration, and eradication of infection leading to satisfactory functional outcomes.

## Introduction

Open tibial fractures are a significant concern, primarily resulting from high-energy trauma such as road traffic accidents (RTAs). These injuries predominantly affect younger individuals and are often associated with extensive soft tissue loss, frequently leading to Gustilo-Anderson Type IIIB fractures. Since the classification system was first introduced by Gustilo and Anderson in 1976 [1] and later refined in 1984 [2] into subtypes Type IIIA, Type IIIB, and Type IIIC, the fundamental principles of open fracture management have remained largely consistent.

The cornerstone of open fracture management includes the timely administration of antibiotics, accurate assessment of the injury and wound, and meticulous debridement, all aimed at preventing deep infections. In Type IIIB fractures, soft tissue coverage is equally crucial and should be performed as early as possible. The planning for coverage depends on several factors, including wound readiness, the fixation strategy, and the availability of expertise and resources.

Extensive research has established benchmarks for managing these injuries in the acute setting, emphasizing early intervention for Type IIIB fractures requiring soft tissue coverage. The American College of Surgeons Trauma Quality Improvement Program recommends completing coverage within seven days for wounds requiring skin grafts or soft tissue transfers [3]. In contrast, the British Orthopaedic Association Standards for Trauma (BOAST-4) advises definitive coverage within 72 hours if it cannot be performed during the initial debridement [4].

Pincus et al. [5], in a large multicenter study involving 672 patients, confirmed that delaying coverage beyond seven days increased the risk of deep infections and osteomyelitis by 40% for each additional week of delay. Kuripla et al. [6] reported that the time from definitive fixation to flap coverage was a critical predictor of infection, with delays beyond seven days significantly increasing the risk. 

Despite strong evidence supporting early soft tissue coverage, adherence to these timelines remains inconsistent. Pincus et al. [5] noted that over 60% of patients in North America did not receive flap coverage within the recommended seven-day period. Contributing factors include logistical barriers, limited access to multidisciplinary teams, and variations in institutional practices.

In conclusion, delayed coverage is consistently associated with increased infection rates, prolonged healing times, and a higher risk of complications. 

Despite the extensive literature on acute Gustilo-Anderson Type IIIB open tibial fractures, few studies specifically address old Type IIIB fractures. We define an old Gustilo-Anderson Type IIIB open tibial fracture as a Type IIIB open tibial fracture that, despite initial debridement and skeletal fixation, presents beyond three weeks with deep infection, superinfection, and has not yet undergone definitive soft tissue coverage, regardless of wound size.

Aims and objectives

This study analyses the outcomes of old Gustilo-Anderson Type IIIB open tibial fractures managed using a medial gastrocnemius flap or a combined medial gastrocnemius-hemisoleus flap, supplemented by a specific protocol to control infection and promote bone healing. It focuses on infection control, wound bed optimization, and definitive coverage using these flap techniques, evaluating their effectiveness in achieving durable soft tissue coverage, infection eradication, fracture union, and bone graft incorporation.

## Materials and methods

Study design

This was a retrospective descriptive study involving 15 participants with old Gustilo-Anderson Type IIIB open tibial fractures. The study period spanned from January 2019 to September 2024. The study has been approved by the Institutional Ethics Committee of ICARE Institute of Medical Sciences and Research, Haldia, West Bengal, India.

Study participants

All 15 cases in this study were initially managed at peripheral centres with antibiotics, single-stage debridement, and fracture fixation using an external fixator. In the primary setting, no definitive procedure was performed for soft tissue coverage in any of these cases. The patients presented to us for further management between 1-3 months post-injury with established deep infections and superinfection. Therefore, we classified these cases as "old Gustilo-Anderson Type IIIB open tibial fractures." All 15 tibial fractures were associated with concurrent fibular fractures. Notably, fracture comminution was minimal, with only four cases having butterfly fragments involving less than one-third of the tibial circumference.

Exclusion criteria

Patients with soft-tissue defects or fractures of the lower third of the tibia were excluded from the study. Additionally, cases with segmental bone loss or bony comminution/butterfly fragmentation involving more than one-third of the tibial circumference were also excluded. This was done to ensure the homogeneity of cases for a more accurate assessment of effectiveness. 

Management protocol

An old Gustilo-Anderson type IIIB tibial fracture presented as a deeply infected and contaminated (superinfection) wound with acute osteomyelitis of the tibia. Our management protocol was designed to first control the active infection (both deep infection and superinfection) through daily irrigation, administration of culture-sensitive antibiotics, and wound debridement. This was followed by the definitive stage, which involved decorticating the exposed tibia, refreshing the fracture ends to assess vascularity, and debriding the outer cortical bone to promote revascularisation. Revision external fixation was performed, and cancellous bone grafts were placed around the fracture site to fill any gaps. Finally, a medial gastrocnemius or combined medial gastrocnemius-hemisoleus flap procedure was performed to enhance revascularisation of the devascularised tibia, promote bony union, and eradicate the infection.

The above idea was supported by an experimental study conducted on dogs by Richards et al. in 1989, which documented the revascularisation effects of muscle flap coverage on bone blood flow following the devascularisation of a segment of the tibia [7]. It was also supported by a clinical study by Nejedlý et al. [8]. The affected limbs exhibited oedema and swelling, particularly in the leg and foot. A deep wound swab/collection was sent for Gram staining along with a culture and sensitivity test upon admission, and empirical antibiotics were administered parenterally.

Wound management

The wound was irrigated with copious saline under strict aseptic conditions, ensuring thorough irrigation of deep pockets. A saline-soaked gauze was applied, followed by a well-padded sterile dressing. This daily regimen of irrigation and sterile dressing continued until the wound was ready for preliminary debridement. 

Once the culture and sensitivity reports were available, specific antibiotics were administered parenterally, alongside daily wound irrigation. Infection control was achieved within 6-10 days, as indicated by reduced leg oedema, foot swelling, wrinkle signs, and improved inflammatory markers. 

During the preliminary debridement, a systematic approach and careful handling of deep spaces were used to delineate healthy tissue. Deep wound swabs were collected for culture, and the wound was irrigated in stages. Post-debridement, the wound was left open, covered with saline-soaked gauze, dressed with a sterile dressing, and changed daily.

Definitive Soft Tissue Coverage With Medial Gastrocnemius / Combined Medial Gastrocnemius-Hemisoleus Flap

In all 15 cases, the external fixators were removed, and the wounds were irrigated with saline, followed by fresh dressings and draping of the entire limb, including the ipsilateral iliac crest. Saline irrigation continued in stages throughout the procedure. The wound was scraped of granulation tissue, and exposed tibial fracture ends were carefully examined, discarding any loose fragments without soft tissue attachment. In all cases, at least two-thirds of the tibial circumference was intact, allowing proper reduction and fixation. Four cases had butterfly fragments involving less than one-third of the circumference, which were allowed to fall on their places with the primary fragments. 

The tibial fragments were decorticated circumferentially with sharp osteotomes under saline irrigation, but no punctate bleeding points were observed up to 2 cm to 4 cm from the fracture ends. The fracture ends were refreshed with osteotomes but showed no bleeding. The fractures were anatomically reduced and stabilised with a unilateral uniplanar AO tubular fixator, ensuring proper placement of Schanz screws to avoid interference with the flap. Compression was applied using the fixator to ensure optimal contact and rigidity. 

Free cancellous bone grafts were harvested from the ipsilateral iliac crest and placed around the fracture site to promote healing. A medial longitudinal incision was made to mobilise the medial gastrocnemius/combined medial gastrocnemius and hemisoleus flap, which was raised and positioned to cover the bone and graft without tension, obliterating any dead space. A split-thickness skin graft was harvested from the ipsilateral thigh and applied over the flap. Standard dressings were applied, and the limb was kept elevated. The first wound inspection and dressing change occurred on the fifth postoperative day, with subsequent wound management following standard protocol until complete soft tissue healing.

Secondary Debridement Following Flap Cover

In some instances, a discharging sinus with granulation tissue developed at the flap corners post-operatively. Exploration revealed the sinuses were linked to the deep part of the fracture.

During the procedure, great care was taken to preserve the flap, which had become firmly adherent to the tibia. Minimal elevation of the flap revealed punctate bleeding points in areas previously decorticated, where no bleeding had been observed initially. Debridement was performed and adjacent soft tissue was adjusted to obliterate dead space. The wound was closed with a mini negative-pressure drain, which was removed after a few days. Culture reports were reviewed to confirm consistency with prior results

Minor complications, including partial loss of the skin graft and flap tip necrosis, were managed with standard methods.

Antibiotic therapy and follow-up

Specific antibiotics were continued for 3-5 weeks after wound healing, until blood parameters (ESR and CRP) returned to normal, and no clinical signs of infection were present. Regular monitoring was conducted for any adverse effects of prolonged antibiotic use. 

The follow-up period ranged from 1 year and 3 months to 6 years and 1 month.

## Results

The patients' ages ranged from 25-54 years, with a mean age of 39.6 years. The cohort comprised 12 male and 3 female patients. The interval between the initial injury and presentation to our centre ranged from 1 month and 8 days to 3 months and 4 days, with an average of 2 months and 7 days. The time required to optimise wound conditions for the definitive stage ranged from 10 to 16 days, with a mean of 13.73 days.

Table 1 presents a detailed overview of the demographic data, the variables associated with the cases, the duration of the follow-up period, and the complications observed.

**Table 1:** Demographic data, variables of cases, follow-up period, and complications.

Case no	Age in years	Gender/occupation/side	Wound size (soft-tissue loss over tibia)/ Length of exposed tibia/ length of devascularised tibia	Time gap since injury	Time taken to optimise the wound for flap cover	Secondary procedure required after soft tissue cover with a time gap	Time taken for fracture union since injury	Follow-up period in the study
Case 1	45	Male / Manual labour / Right	12 cm x 8 cm / 11 cm / 7 cm	One month and 18 days	12 days	Secondary debridement at 42 days	Nine months	One year and three months
Case 2	25	Male / Outdoor office job / Right	10 cm x 8 cm / 10 cm / 5 cm	Two months and 12 days	11 days	NIL	Eight months	One year and seven months
Case 3	34	Male / Manual labour / Left	14 cm x 9 cm / 12 cm / 5 cm	One month and 26 days	10 days	Flap tip necrosis treated conservatively	Six months	One year and 11 months
Case 4	38	Male / Car driver / Right	11 cm x 10 cm / 10 cm / 4 cm	Two months and 9 days	14 days	Partial loss of skin graft treated conservatively	Eight months	Two years and one month
Case 5	29	Female / Domestic worker/ Left	12 cm x 10 cm / 11 cm / 7 cm	One month and 23 days	15 days	NIL	Nine months	Two years and three months
Case 6	48	Male / Manual labour / Right	10 cm x 8 cm / 10 cm / 7 cm	One month and 8 days	12 days	NIL	Eight months	Two years and eight months
Case 7	37	Male / Fruit seller / Right	13 cm x 9 cm / 12 cm / 8 cm	Two months and 6 days	14 days	NIL	Ten months	Two years and nine months
Case 8	44	Female / Domestic worker / Right	15 cm x 8 cm / 14 cm / 7 cm	One month and 25 days	13 days	NIL	Nine months	Two years and 11 months
Case 9	28	Male / Manual worker / Left	13 cm x 10 cm / 12 cm / 4 cm	Two months and 16 days	15 days	Partial loss of skin graft treated conservatively	Six months	Three years and six months
Case 10	49	Male / Manual labour / Right	14 cm X 10 cm / 12 cm / 4 cm	Two months 23 days	14 days	NIL	Eight months	Three years and 11 months
Case 11	54	Female / Caregiver in an old-age home / Right	14 cm X 9 cm / 13 cm / 5 cm	Two months and 12 days	15 days	Partial loss of skin graft treated conservatively	Nine months	Four years and three months
Case 12	37	Male / Vendor / Right	12 cm X 9cm / 11 cm / 4 cm	One month and 23 days	14 days	NIL	Seven months	Four years and six months
Case 13	36	Male / Car driver / Left	10 cm X 10 cm / 10 cm / 4 cm	Two months and 28 days	16 days	NIL	Eight months	Four years and 11 months
Case 14	52	Male / Outdoor office job / Left	11 cm X 9 cm / 10 cm / 4 cm	Two months and 21 days	15 days	NIL	Nine months	Five years and six months
Case 15	38	Male / Manual worker / Right	13 cm X 8 cm / 12 cm / 5 cm	Three months and 4 days	16 days	Secondary debridement at 62 days	Nine months	Six years and one month

A total of 14 injuries resulted from high-energy trauma due to RTAs, while one case involved a high-velocity heavy machinery accident at a construction site. None of the patients had associated injuries involving other parts of the body, nor did they have any uncontrolled comorbid conditions. The majority of fractures were primarily located in the middle third of the tibia. None of the fractures or associated wounds extended distally beyond the middle third (cut-off level), though some had extensions into the proximal third. All cases underwent primary skeletal stabilization using AO tubular external fixation. The affected bone in these cases was the tibial cortical diaphysis (shaft of the tibia). The soft-tissue defects measured between 10-15 cm in length and 8-10 cm in width. All cases presented with unhealthy wounds characterized by frank purulent discharge, with the length of the exposed tibia ranging from 10-14 cm (Table 1). 

The viability of the bone was assessed using punctate bleeding points during decortication. A segment of 2-4 cm at each end of the cortical tibia showed no bleeding, indicating devascularisation. The total length of devascularised tibia ranged from 4-8 cm (Table 1).

All 15 cases achieved bony union without signs of infection, as confirmed both radiologically and clinically at the follow-up. The time to tibial union ranged from 6-9 months after injury, with successful incorporation of bone grafts. 

Normal joint movements of the knee, ankle, and foot were restored in all cases. Gait was normalized, and all patients returned to their previous occupations without any disability in daily activities. The strength of the triceps surae on the affected side remained comparable to that on the unaffected side.

Complications

One case experienced minor superficial flap tip necrosis, which was debrided and healed with dressings. In three cases, partial-thickness skin grafts had partial skin loss, which was managed with regular dressings, leading to adequate coverage through epithelialisation. All reconstructed soft tissue coverage was thick, stable, and exhibited a near-normal texture.

In two cases, infections developed at 1.5 months and 2 months following flap coverage. Both were successfully managed with secondary debridement, which effectively controlled the infection; the culture reports were consistent with previous findings. 

Apart from these two cases, no further infections were observed, and all 15 cases remained infection-free at the final follow-up.

Case 1

The next four images illustrate the journey of Case No. 1, a patient with a one-and-a-half-month-old Gustilo-Anderson Type IIIB open tibial fracture in the mid-third tibia. This case highlights various stages, including findings at presentation, intraoperative, and postoperative findings, as well as follow-up outcomes.

**Figure 1:**
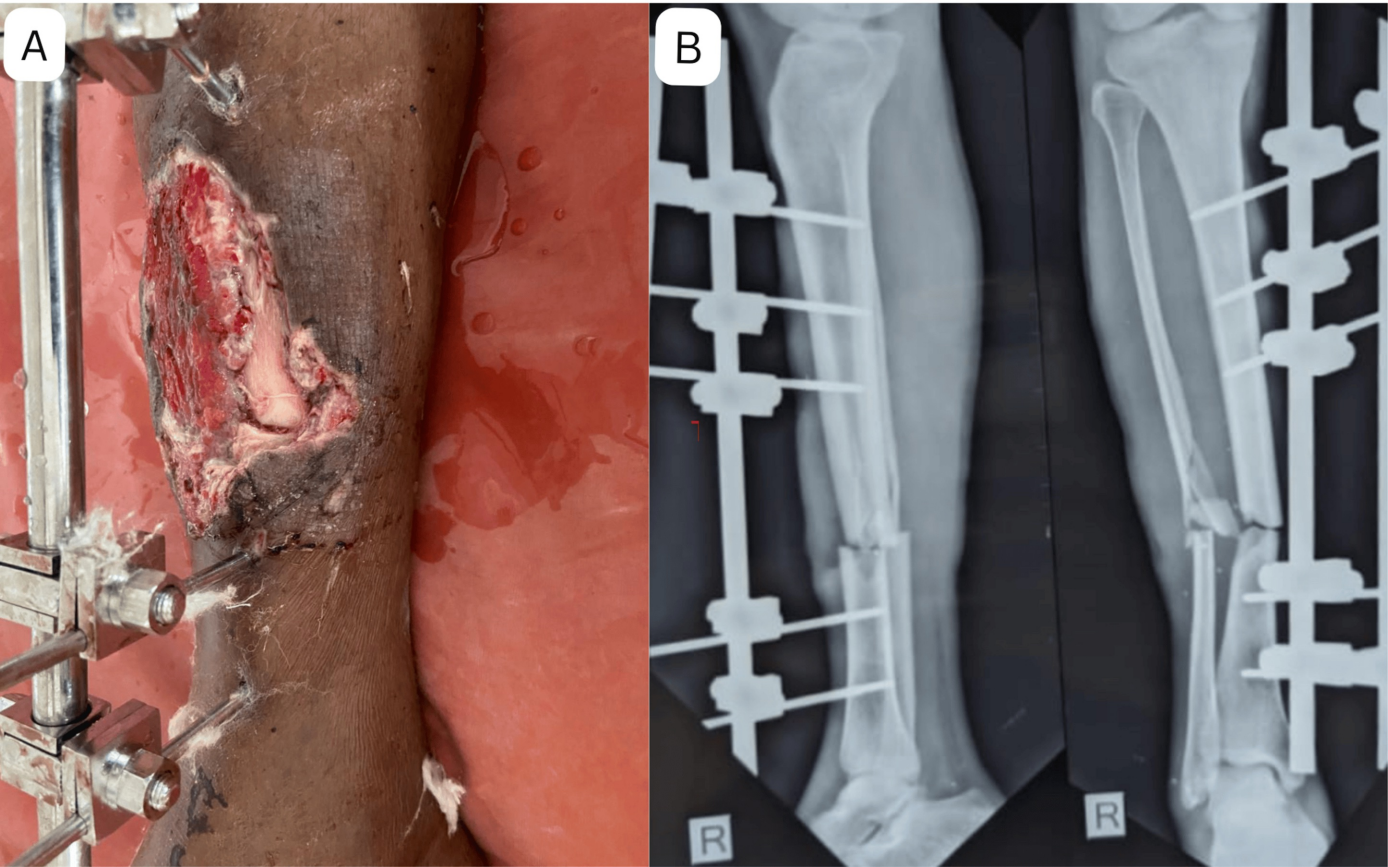
One-and-a-half-month old Gustilo-Anderson Type IIIB open tibial fracture on presentation. (A) Infected wound in mid third tibia, exposed tibia with gap at fracture site with AO tubular fixator in-situ. (B) Anteroposterior and lateral radiograph of both bones leg. These images correspond to Case 1 from the study.

Figure 1 shows the initial presentation of the case. Panel (A) depicts an infected wound in the mid-third of the tibia with exposed bone and a fracture gap, accompanied by an AO tubular fixator in situ. Panel (B) displays the anteroposterior and lateral radiographs of the leg, revealing the extent of the fracture, a butterfly fragment, and a gap at the fracture site. 

**Figure 2:**
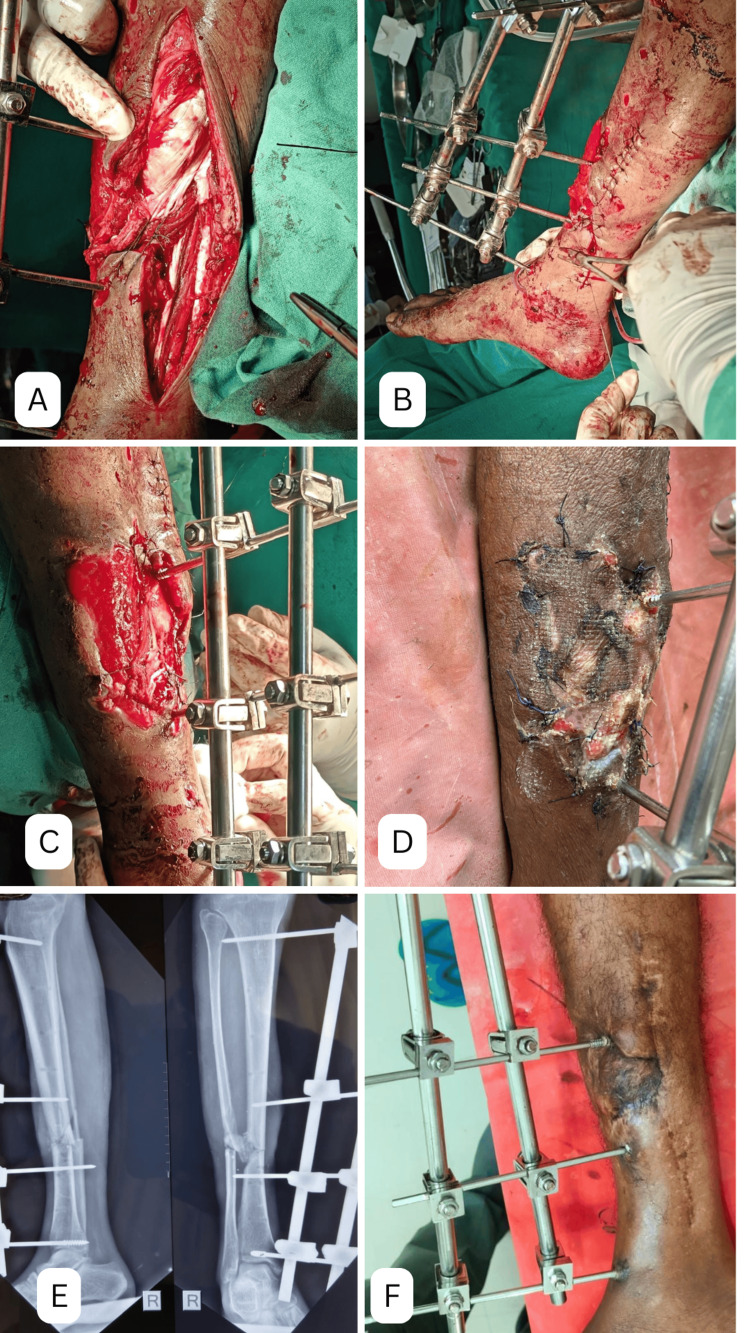
(A) Combined medial gastrocnemius and hemisoleus flap cover. (B) Closure of medial incision for taking flap. (C) Bone covered with combined medial gastrocnemius and hemisoleus flap and granulation tissue over anterior compartment muscle prior to putting a split skin graft. (D) Split skin graft applied over flap and granulation tissue (post-operative day 5). (E) Immediate post operative anteroposterior and lateral radiographs. (F) Flap cover at six weeks. These images correspond to Case 1 from the study.

Figure 2 illustrates the surgical steps and early postoperative outcomes. Figure 2A shows the combined medial gastrocnemius-hemisoleus flap used to cover the exposed tibia. Panel (B) captures the closure of the medial incision made to harvest the flap, while Figure 2C demonstrates the tibia covered with the combined medial gastrocnemius-hemisoleus flap and granulation tissue forming over the anterior compartment muscle just before a split-thickness skin graft was applied. Panel (D) depicts the application of the graft on postoperative Day 5. Figure 2E-2F presents the immediate postoperative anteroposterior and lateral radiographs and the flap cover at six weeks, respectively.

**Figure 3:**
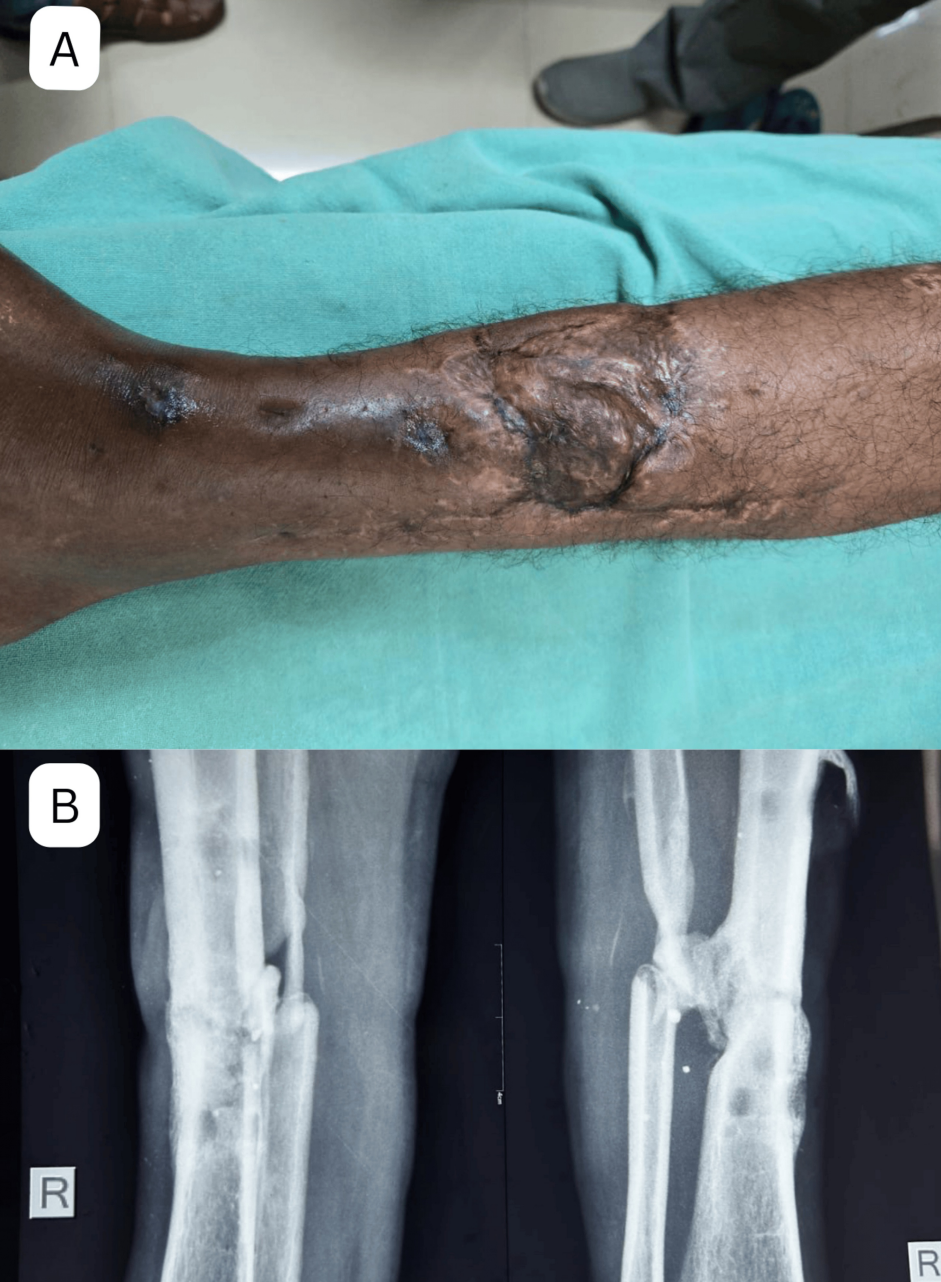
(A) Flap cover at seven months after removal of external fixator. (B) Anteroposterior and lateral radiograph at seven months since definitive procedure follow-up (nine months since injury) showing union of tibia, incorporation of bone graft with no sign of active infection. These images correspond to Case 1 from the study.

Figure 3A shows the appearance of the flap following the removal of the external fixator (seven months follow-up in the study). Figure 3B features radiographs taken at the same follow-up period (seven months since the definitive procedure, nine months since injury) demonstrating the union of the tibia, incorporation of the bone graft, and an absence of active infection. 

**Figure 4:**
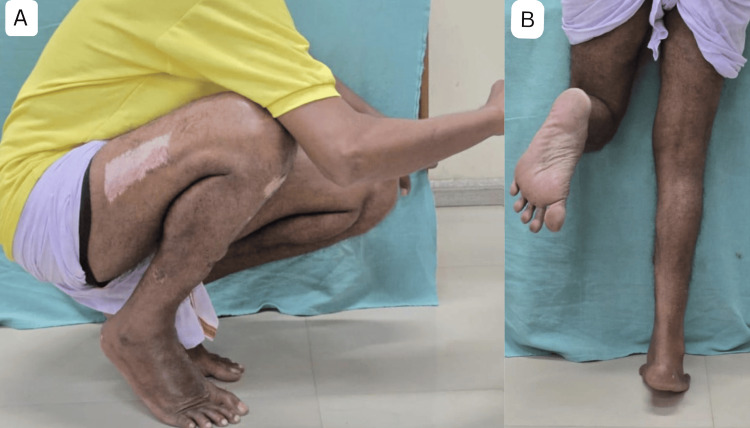
Functional outcome at nine months follow-up. (A) Squatting on heels. (B) Standing on tip-toes of affected side. These images correspond to Case 1 from the study.

Figure 4 highlights the functional outcomes at nine months. Figure 4A depicts the patient’s ability to squat on heels, illustrating the range of motion in the knee, ankle, and foot. Figure 4B showcases the strength of the triceps surae muscle, as evidenced by the patient standing on tip-toes on the affected side.

Wound culture results

Upon admission, a deep wound swab/collection was sent for Gram staining along with a culture and sensitivity test, which identified *Staphylococcus aureus*, *Escherichia coli*, *Streptococcus* spp., *Klebsiella* spp., *Proteus* spp., and *Pseudomonas* spp. The most common isolates were* Staphylococcus aureus* (six cases) and* Escherichia coli* (four cases).

The isolated bacteria showed sensitivity to third-generation cephalosporins (e.g., ceftriaxone, cefotaxime, ceftazidime), linezolid (an oxazolidinone antibiotic), aminoglycosides (e.g., gentamicin, amikacin), and meropenem (a carbapenem). While some bacteria were resistant to multiple antibiotics, they remained sensitive to certain others.

After debridement was conducted prior to the definitive stage, cultures were taken again. Of the 15 wounds, 14 cultures were negative, while one remained positive, consistent with the previous report.

## Discussion

The management of Gustilo-Anderson Type IIIB open tibial fractures requires a multidisciplinary approach due to the high-energy nature of the injury and significant soft tissue loss, which increase the risk of infection, nonunion, and functional deficits. Key elements include timely antibiotic administration, thorough debridement, appropriate fracture stabilisation, and early soft tissue coverage.

A thorough understanding of reconstructive options for soft tissue loss in Gustilo-Anderson Type IIIB fractures is essential. The reconstructive ladder provides guidance in selecting the most appropriate coverage, emphasising the principle of choosing the least complex option that ensures the best functional outcome [9].

In contrast to acute injury, the primary challenge in an old Gustilo-Anderson Type IIIB open tibial fracture was a deeply infected wound with acute osteomyelitis and superinfection (contamination from the external environment). Our management protocol aimed to control the infection through daily irrigation, antibiotics, and wound debridement to reduce the bacterial load, thereby optimising the wound for definitive management. This was followed by a definitive procedure as described above. The use of muscle flap coverage in our approach is supported by an experimental study by Richards et al. (1989), which demonstrated that muscle flaps promote bone blood flow [7], as well as a clinical study by Nejedlý et al. [8].

Since superinfection is often caused by different, typically more resistant microorganisms, we relied on daily wound irrigation under strict aseptic conditions and debridement to reduce the microbial load and optimise the wound. Although a period of 10 to 16 days may seem excessive for wound preparation before the definitive procedure, we believe that extending this timeframe for heavily infected wounds (deep infection and superinfection) is justified to achieve long-term success. Mild to moderately infected wounds require less time, as they can be assessed clinically and monitored using hematological markers.

A noteworthy observation during secondary debridement in two cases (at 42 and 62 days post-flap coverage) was that the muscle flap was firmly attached to the tibia. Upon elevating the flap during debridement, punctate bleeding points were observed at the fracture site, where they had been absent during the initial surgery. This finding strongly suggests that the muscle flap contributes to the vascularisation of the tibial cortex, thereby supporting the healing process.

The medial gastrocnemius or combined medial gastrocnemius-hemisoleus flap not only provided effective soft tissue coverage but also contributed to infection eradication and promoted fracture healing by revascularising the devascularised portion of the tibia and enhancing bone graft incorporation through improved tissue vascularity. This was further confirmed by findings during secondary wound exploration.

In this study, wounds ranged from 10-15 cm × 8-10 cm and were successfully covered using the medial gastrocnemius or combined medial gastrocnemius-hemisoleus flap. A free flap was not considered in this study because all 15 wounds, in both size and location, were suitable for the local muscle flap used. However, a free flap remains the gold standard for covering large defects due to its superior vascularisation potential. Another advantage of the muscle flap is that it conforms to the devascularised bone surface and obliterates dead space. 

The gastrocnemius myocutaneous flap was first described, along with its detailed anatomical vascular supply, by McCraw at the Symposium of Military Plastic Surgery in 1976 and published in 1978 [10]. Feldman et al. described the medial gastrocnemius myocutaneous flap in 1978 [11], supported by an extensive vascular anatomical study.

The medial gastrocnemius flap, supplied by the medial sural artery, is easily mobilised and rotated due to its independent vascular supply. This well-documented flap can be performed with basic surgical skills. The medial half of the soleus is supplied by two to three extra muscular branches (EMBs) from the posterior tibial artery and occasionally from the popliteal artery, while the lateral half receives blood from the peroneal artery. The proximal 25% of the soleus receives EMBs from all three arteries [12]. This understanding allows for the use of a hemisoleus flap. The medial hemisoleus flap is the preferred treatment for soft tissue defects in the middle third of the tibia, offering enhanced vascularity and better infection control [13].

For soft tissue defects in the middle and distal thirds of the leg, in open tibial fracture and/or osteomyelitis, soleus muscle flap is effective and feasible with minor donor site morbidity [14]. For larger defects, the medial gastrocnemius combined with the hemisoleus can provide effective coverage [15].

We also observed these findings in the present study:

The time required for complete fracture union in all cases ranged from 6-10 months after the injury. Patients presented between one and three months post-injury, and fracture reductions were revised with refreshening of the fracture ends and revision fixation. Therefore, the time for union was calculated to be between three and a half and seven and a half months. The prolonged healing period was justified, as all 15 cases involved devascularised tibial segments measuring 4-8 cm, requiring additional time for revascularization. In contrast, excising the devascularised segments and utilising the Masquelet technique, bone transport, or vascularised fibular grafting would have significantly prolonged the treatment duration while also increasing procedural complexity and morbidity.

Gait analysis showed that patients did not experience donor site morbidity or any disability in their average daily activities. All of them returned to their pre-injury status; however, some may face challenges with more strenuous activities, such as brisk walking or walking uphill [16]. 

In this study, we have demonstrated that aggressive interventions targeting both the bone and soft tissue components can achieve optimal outcomes, even in older Gustilo-Anderson Type IIIB open tibial fractures. The limitations of this study include a small sample size, the absence of a control group, and limited literature on older cases. No PROM scoring system was used, as we were unable to match the participants' lifestyles and activity levels to a standard scoring system. Consequently, functional outcomes were measured subjectively. A multi-institutional study comparing various methods would provide more robust scientific data and statistical analysis.

## Conclusions

The protocol used to manage deep wound infection with superinfection and acute osteomyelitis of the tibia in old Gustilo-Anderson Type IIIB fractures proved effective through both bony management and the medial gastrocnemius or combined medial gastrocnemius-hemisoleus flap procedures. The local muscle flap not only provided soft tissue coverage but also played a crucial role in eradicating the infection and promoting bone union by revascularising the devascularised portion of the tibia and enhancing bone graft incorporation through neovascularisation. This approach yielded satisfactory outcomes despite the delayed presentation with deep infection and superinfection.
